# Development and validation of a nomogram for predicting lung cancer based on acoustic–clinical features

**DOI:** 10.3389/fmed.2025.1507546

**Published:** 2025-01-21

**Authors:** Zhou Lu, Jiaojiao Sha, Xunxia Zhu, Xiaoyong Shen, Xiaoyu Chen, Xin Tan, Rouyan Pan, Shuyi Zhang, Shi Liu, Tao Jiang, Jiatuo Xu

**Affiliations:** ^1^School of Traditional Chinese Medicine, Shanghai University of Traditional Chinese Medicine, Shanghai, China; ^2^Department of Acupuncture and Moxibustion, Huadong Hospital, Fudan University, Shanghai, China; ^3^Department of Thoracic Surgery, Huadong Hospital, Fudan University, Shanghai, China; ^4^School of Computer Science and Technology, East China Normal University, Shanghai, China

**Keywords:** acoustic diagnosis, lung cancer, nomogram, machine learning, lasso regression algorithm

## Abstract

**Objective:**

Lung cancer—with its global prevalence and critical need for early diagnosis and treatment—is the focus of our study. This study aimed to develop a nomogram based on acoustic–clinical features—a tool that could significantly enhance the clinical prediction of lung cancer.

**Methods:**

We reviewed the voice data and clinical information of 350 individuals: 189 pathologically confirmed lung cancer patients and 161 non-lung cancer patients, which included 77 patients with benign pulmonary lesions and 84 healthy volunteers. First, acoustic features were extracted from all participants, and optimal features were selected by least absolute shrinkage and selection operator (LASSO) regression. Subsequently, by integrating acoustic features and clinical features, a nomogram for predicting lung cancer was developed using a multivariate logistic regression model. The performance of the nomogram was evaluated by the area under the receiver operating characteristic curve (AUC) and the calibration curve. The clinical utility was estimated by decision curve analysis (DCA) to confirm the predictive value of the nomogram. Furthermore, the nomogram model was compared with predictive models that were developed using six additional machine-learning (ML) methods.

**Results:**

Our acoustic–clinical nomogram model demonstrated a strong discriminative ability, with AUCs of 0.774 (95% confidence interval [CI], 0.716–0.832) and 0.714 (95% CI: 0.616–0.811) in the training and test sets, respectively. The nomogram achieved an accuracy of 0.642, a sensitivity of 0.673, and a specificity of 0.611 in the test set. The calibration curve showed excellent agreement between the predicted and actual values, and the DCA curve underscored the clinical usefulness of our nomogram. Notably, our nomogram model outperformed other models in terms of AUC, accuracy, and specificity.

**Conclusion:**

The acoustic–clinical nomogram developed in this study demonstrates robust discrimination, calibration, and clinical application value. This nomogram, a unique contribution to the field, provides a reliable tool for predicting lung cancer.

## 1 Background

Lung cancer has emerged as the leading cause of death among individuals with malignant tumors (1), exhibiting a gradual annual increase in both incidence and mortality rates. Numerous epidemiological studies have indicated that the high incidence of lung cancer is closely associated with smoking (2), genetic susceptibility (3), the aging population (4), and exposure to carcinogenic substances (5, 6), such as the growing environmental pollution and secondhand smoke. Early detection, accurate diagnosis, and timely treatment are crucial for improving the survival rate of lung cancer patients.

Current clinical studies have reported various methods for early lung cancer screening and diagnosis, including computed tomography (CT) imaging diagnosis, serum tumor markers detection, and sputum cytology examination. Low-dose computed tomography is considered the preferred screening method for early-stage lung cancer (7). However, it remains controversial (8) due to concerns such as false-positive results, overdiagnosis and overtreatment, radiation dose, and the cost–benefit ratio of screening. Early-stage lung cancer typically manifests as a pulmonary nodule on CT imaging, and its pathological nature requires confirmation through pathological tissue obtained through invasive methods such as surgery or percutaneous lung biopsy. Therefore, achieving high-accuracy diagnosis and classification of lung cancer through non-invasive examination methods has emerged as a focal point of research, to reduce patient harm. In addition, there is a pressing need to develop convenient and effective screening methods for lung cancer in settings where access to extensive medical equipment is constrained.

Auscultation, a vital diagnostic technique in clinical practice, enables physicians to detect diseases by using their auditory perception to interpret patient’s pathological sounds and speech. However, the classical acoustic diagnosis still faces numerous challenges, including the lack of objective diagnosis results from individual auditory differences and the confusion caused by noise in the diagnostic environment. In recent years, with the development of speech recognition technology, more methods have been introduced to facilitate the objective analysis of auscultation. Modern acoustic diagnosis involves collecting patients’ voices through hardware devices such as microphones and utilizing computer technology to qualitatively and quantitatively analyze these voice signals, ultimately yielding objective and informative diagnostic outcomes.

Acoustic diagnosis—with its simplicity, speediness, and non-invasiveness—plays a pivotal role in the initial evaluation and monitoring of respiratory conditions. With the developments in sensor technology and computational analysis methods, it has become possible to measure and interpret internal lung acoustic signals, such as breathing or vocal sounds (9). Yan et al. (10) applied sample entropy for wavelet packet transform coefficients to quantify the signals from three patterns of traditional Chinese medicine and achieved higher than 90% recognition accuracy rates with a support vector machine. Song et al. (11) explored the phonetic characteristics of patients with pulmonary nodules (PNs) and found that there were statistically significant differences in pitch, intensity, and shimmer in patients with PNs compared with healthy people, and PNs with diameters ≥8 mm had a significantly higher third formant. Porter et al. (12) developed an automatic cough detector and applied a Time Delay Neural Network to identify asthma, pneumonia, lower respiratory tract disease, croup, and bronchiolitis in children. In recent years, the outbreaks of infectious diseases and imbalanced medical conditions have urgently required the development and application of telemedicine. The application of voice signals collected by microphone devices can serve as a convenient and effective tool for remote diagnosis and screening of disease. The research study by Asiaee et al. (13) revealed significant differences in acoustic parameters of sustained vowel “a” in COVID-19 patients compared to healthy subjects. Pahar et al. (14) used audio recordings to detect COVID-19 through transfer learning and bottleneck feature extraction, and results showed that the ResNet50 classifier performed best on all datasets, with areas under the receiver operating characteristic (ROC AUCs) of 0.98, 0.94, and 0.92, respectively, for all three sound classes: coughs, breaths, and speech. However, the research on acoustic diagnosis of lung cancer remains to be explored.

Our previous research focused on analyzing changes in frequency features, energy/amplitude features, and spectral features between patients with PNs and healthy individuals. In this study, we followed up on the pathological examination results of patients with pulmonary lesions and developed a machine-learning (ML) prediction model based on acoustic–clinical features, aiming to provide auxiliary technology for clinical diagnosis and screening of lung cancer.

## 2 Materials and methods

### 2.1 Participants

We recruited volunteers and collected data from the Department of Thoracic Surgery at Huadong Hospital, affiliated with Fudan University, from October 2022 to November 2023. Institutional ethics committees approved the study.

The inclusion criteria for pulmonary lesions are as follows: (a) The patient must be aged between 30 and 80. (b) patients who had undergone CT thorax scans and were found to have at least one pulmonary nodule or mass, which was highly suspicious of malignancy based on radiological criteria, were scheduled for further pathological examinations. (c) The patients should not have received any prior treatment for pulmonary conditions, including inhalation therapy, bronchoscopy, transbronchial biopsy, or thoracic surgery, prior to collecting their voice samples and must demonstrate good compliance. At the same time, healthy volunteers aged between 30 and 80 years with no significant abnormalities found on lung CT scans were included. Furthermore, we excluded (a) patients who had been diagnosed with lung cancer, chronic obstructive pulmonary disease, pulmonary embolism, or tuberculosis before sampling, or those who had a history of acute respiratory infection within the past three months; (b) patients with severe cardiac, cerebral, hepatic, or renal dysfunction; (c) psychiatric patients; (d) patients with auditory, speech, or cognitive impairment; (e) patients with a history of neck surgery, throat surgery, tracheostomy, thyroidectomy, as well as those who had vocal cord paralysis due to non-lung cancer factors such as trauma, neck tumors, central nervous system diseases, infections, or drug side effects; (f) pregnant or lactating women; (g) non-native Chinese speakers.

**Figure 1:**
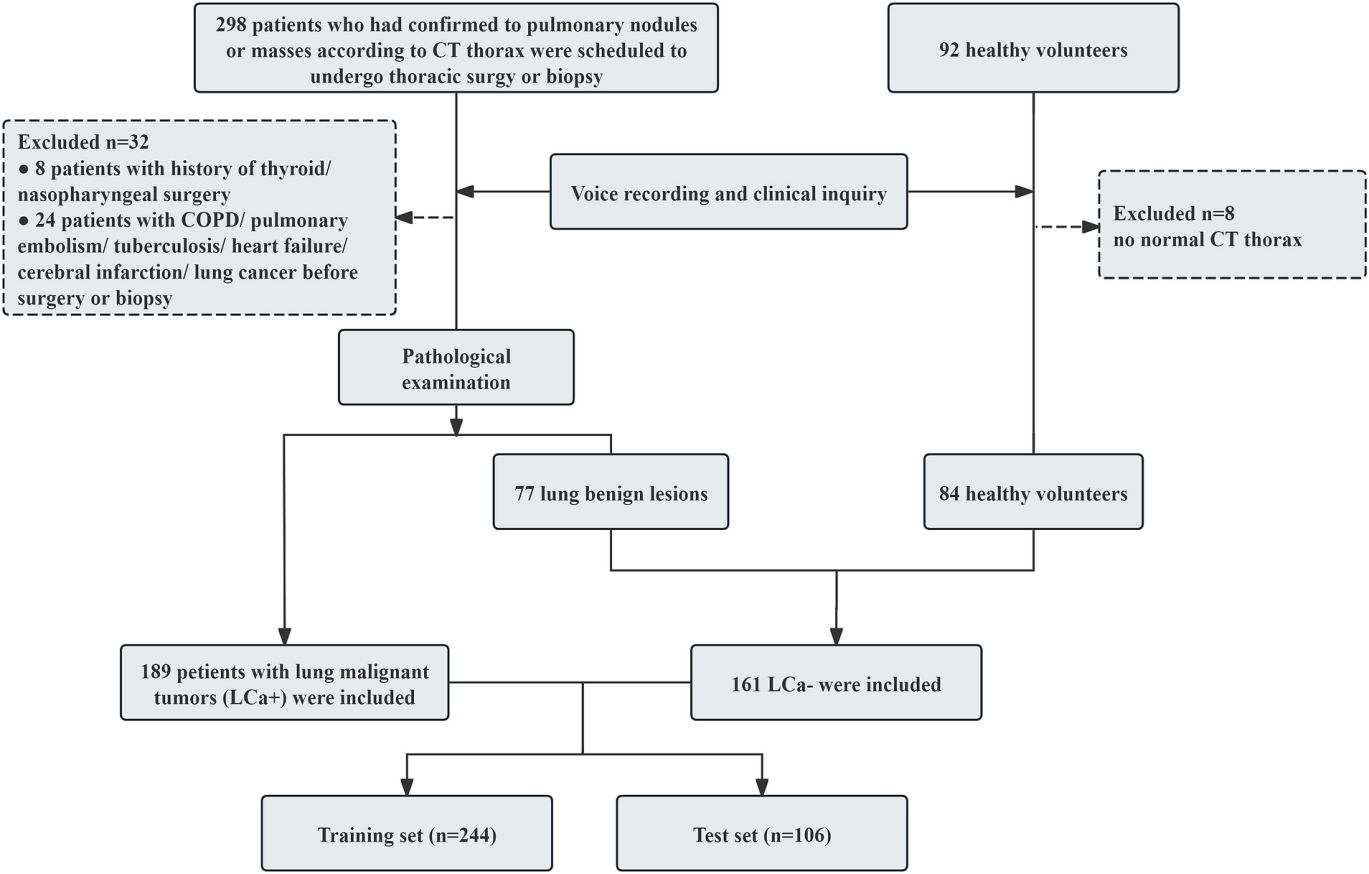
Flowchart depicts the participants’ enrollment process in the study.

According to the pathological examination results, 189 patients with lung cancer (LCa+) and 161 participants with non-lung cancer (LCa−), which included 77 patients with benign lung lesions, and 84 health volunteers were included in this study. The participants’ enrollment process is illustrated in Figure 1.

### 2.2 Procedure

#### 2.2.1 Clinical data collection

Through inquiries, we recorded the clinical data of all participants, including age, sex, family history of cancer, smoking history (with a smoking index ≥400 cigarette-years), and eight clinical symptoms experienced within the past month: cough, expectoration, chest tightness/pain, asthma, fatigue, insomnia, abnormal sweating, and susceptibility to colds (suffering from colds ≥3 times per year). Prior to collection, all participants had signed informed consent forms.

#### 2.2.2 Voice recording

Sony A10 linear pulse-code modulation (PCM) recording device was used to collect voice signals from the participants. Voice recordings were conducted on patients with pulmonary nodules or masses before surgical intervention or biopsy. The noise level in the sampling environment was maintained below 45 decibels to ensure recording quality. The device was placed 10 cm from the participant’s mouth, inclined at 45°. The sampling rate was set at 44.1 kHz, with a bit depth of 16 bits, to ensure high-fidelity audio recording. Participants were instructed to produce, with a comfortable and steady pitch and constant amplitude, the five Mandarin Chinese vowels ([a], [e], [i], [o], and [u]), each sustained for 2 s.

#### 2.2.3 Voice signal preprocessing

We utilized the Cool Edit Pro 2.1 software (Syntrillium Software Corporation) for audio editing, manually eliminating noise and redundant information. Additionally, the Praat (version 6.1.51, developed by Paul Boersma and David Weenink, Phonetic Sciences, University of Amsterdam) voice analysis program was also employed for vowel annotation, endpoint detection, and audio segmentation. The middle 0.5-s stable portion of the acoustic signal from each vowel’s acoustic signal was extracted for analysis.

#### 2.2.4 Acoustic features extraction

Acoustic features were extracted with OpenSMILE toolkit (audEERING GmbH, Gilching, Germany) (15), employing the extended Geneva Acoustic Minimalistic Parameter Set (eGeMAPS) (16, 17). The original parameter set comprises physical acoustic parameters (18) (i.e., low-level descriptors) and their statistical functionals. Since this study focused solely on voiced sounds, the voice signal of individual vowels was relatively stable, resulting in the exclusion of unvoiced regions from the analysis. The following features were selected for analysis in this study: ① Frequency features, including fundamental frequency (F0), jitter, formant 1–3 frequency, and formant 1–3 bandwidth; ② energy/amplitude features, including shimmer, loudness, harmonics-to-noise ratio (HNR); ③ spectral parameters, including H1–H2 harmonic difference (H1–H2), H1–A3 harmonic difference (H1–A3), and formant 1–3 relative energy, MFCCs 1–4, spectral flux, alpha ratio, Hammarberg index, spectral slope 0–500 and 500–100 Hz. Audio signals were divided into short frames during preprocessing, and a window function was applied. Given the relative stationarity of speech signals within a short time range, an arithmetic mean was used to calculate each acoustic feature value.

### 2.3 Data preprocessing

All participants were divided into the lung cancer group (LCa+) and the non-lung cancer group (LCa−), based on the pathological results of patients presenting with pulmonary nodules or masses. Age was considered a continuous variable, recorded with actual values. Sex, family history of cancer, smoking history, and the presence of clinical symptoms, including cough, expectoration, chest tightness/pain, shortness of breath, fatigue, insomnia, abnormal sweating, and susceptibility to colds, were all marked as binary variables. To mitigate the influence of differing scales among the indicators, the acoustic feature data were normalized using the min-max normalization method (17), scaled to a range between −1 and 1. In contrast, clinical features, including sex, family history of cancer, smoking history, and clinical symptoms, were maintained in binary format, denoted as either 0 or 1.

### 2.4 Statistical analysis and machine learning

Statistical analysis was conducted using the R software package (version 4.2.1) and Statistical Package for the Social Sciences (SPSS version 26.0, IBM corp). Measurement data adhering to a normal distribution were summarized using mean ± standard deviation (SD), with independent sample *t*-tests conducted for comparison. Conversely, variables that did not follow a normal distribution were described by median (interquartile range), and Mann–Whitney U tests were used for comparison. Categorical variables were compared by chi-square tests. Count data were presented as cases and percentages, with *χ*^2^ tests employed for statistical comparison.

First, the participants were randomly allocated to a training set and a test set in a 7:3 ratio, ensuring that the distribution of outcome events was evenly spread across both sets. The training set served as the basis for screening variables and building the model. Next, the least absolute shrinkage and selection operator (LASSO) regression algorithm (19) was applied to screen the acoustic features. The optimal *λ* was selected using internal 10-fold cross-validation only on the training data, eliminating unimportant acoustic features and retaining those relevant to the identification of lung cancer. A multivariable logistic regression with backward elimination based on the Akaike Information Criterion (AIC) (20, 21) was applied to select independent predictors of lung cancer from the acoustic–clinical features. A nomogram—a widely utilized visual prediction tool in the medical field (22–24)—provides clinicians with a more intuitive and accurate prediction approach. A nomogram for predicting lung cancer was developed based on the logistic regression analysis results. In this nomogram, a score was assigned to each influencing factor based on the magnitude of its regression coefficient, and these scores were visually represented on a graph, forming an intuitive linear diagram corresponding to the diagnostic probability of lung cancer. In addition, we compared the nomogram model with predictive models established using six additional machine learning (ML) methods, including extreme gradient boosting (XGBoost), Adaptive Boosting (AdaBoost), gradient boosting decision tree (GBDT), random forest (RF), support vector machine (SVM), and multilayer perceptron (MLP).

**Figure 2:**
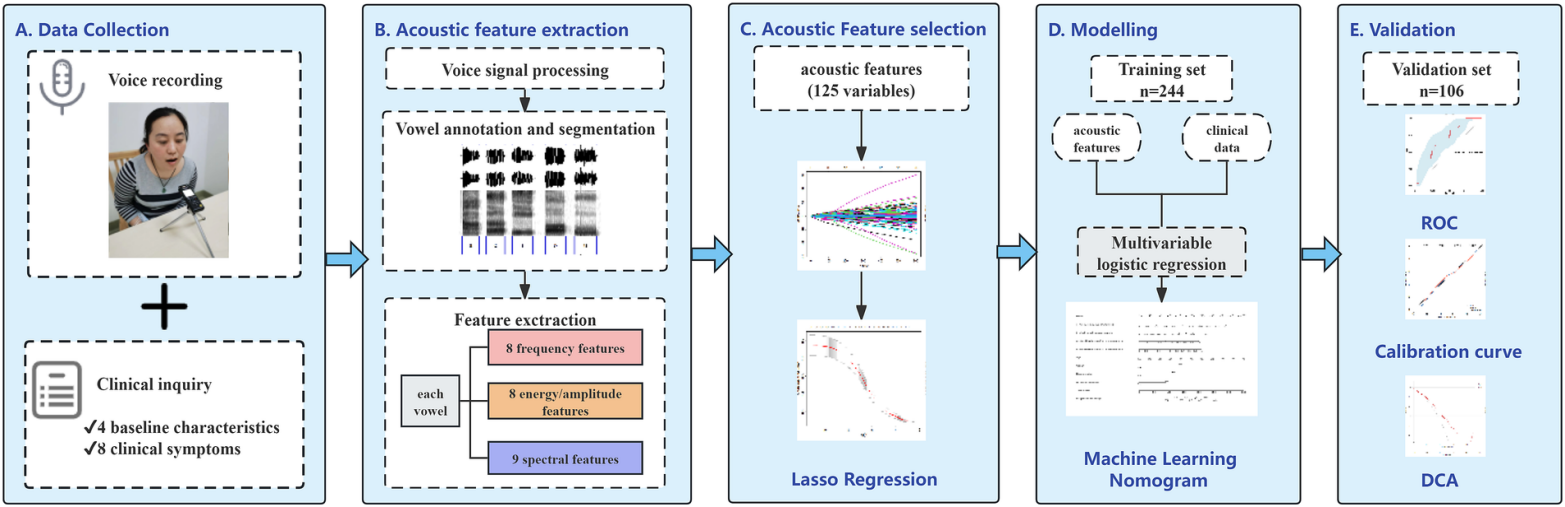
Workflow for development and validation of the proposed nomogram for prediction of lung cancer based on acoustic–clinical features. **(A)** Data collection, collecting voice signals and clinical data from the participants before pathological examination; **(B)** acoustic feature extraction, voice annotation, and segmentation, extracting 25 acoustic features for each vowel; **(C)** classification based on pathological examination results, using LASSO regression to screen lung cancer related acoustic features; **(D)** establishing a model in the training set and developing an acoustic–clinical nomogram for predicting lung cancer; **(E)** performing ROC analysis, calibration analysis, and decision curve analysis in the training set and the test set.

Then, we assessed the generalization of the models by evaluating their predictive performance using the ROC curve, accuracy, sensitivity, specificity, and F1 score. The nomogram’s goodness of fit was assessed through the Hosmer–Lemeshow test and calibration curves. The clinical applicability was also assessed *via* decision curve analysis (DCA), which quantified net benefits at various threshold probabilities. The overall flowchart of the study is shown in Figure 2.

In terms of the R package, the “caret” package was utilized for data grouping and regression training; the “glmnet” package for LASSO regression analysis; the “pROC” package for model development and plotting ROC curves; and the “rms” package for construction of nomogram; and the “rmda” package for DCA. For all analyses, *p* < 0.05 was considered statistically significant, and all tests were conducted with a two-tailed approach.

## 3 Results

### 3.1 Baseline analysis

A total of 350 participants were enrolled in the study cohort, among which 244 cases were assigned to the training set and the remaining 106 to the test set. The clinical and demographic characteristics of the training set and test set are summarized in Table 1. No statistically significant differences were observed in the clinical variables between the two datasets (*p* > 0.05).

**Table 1:** Comparison of clinical data between training set and test set.

Variables	Training set (*n* = 244)		Test set (*n* = 106)		*p-*value^a^
	LCa + (*n* = 137)	LCa − (*n* = 107)	LCa + (*n* = 52)	LCa − (*n* = 54)	
Sex, *n* (%)					0.255
Male	58 (42.3)	47 (43.9)	20 (38.5)	18 (33.3)	
Female	79 (57.7)	60 (56.1)	32 (61.5)	36 (66.7)	
Age (year), median (IQR)	60.0 [52.0,68.0]	54.0 [39.0,64.0]	60.0 [54.0,68.0]	51.0 [39.0,66.8]	0.639
Smoking, *n* (%)					0.351
Active smoker	35 (25.5)	21 (19.6)	17 (32.7)	13 (24.1)	
Non-smoker	102 (74.5)	86 (80.4)	35 (67.3)	41 (75.9)	
Family history of cancer, *n* (%)					0.740
Yes	19 (13.9)	11 (10.3)	6 (11.5)	5 (9.26)	
No	118 (86.1)	96 (89.7)	46 (88.5)	49 (90.7)	
Cough, *n* (%)					0.363
Yes	40 (29.2)	11 (10.3)	10 (19.2)	7 (13.0)	
No	97 (70.8)	96 (89.7)	42 (80.8)	47 (87.0)	
Expectoration, *n* (%)					0.050
Yes	34 (24.8)	8 (7.48)	7 (13.5)	2 (3.70)	
No	103 (75.2)	99 (92.5)	45 (86.5)	52 (96.3)	
Chest tightness/pain, *n* (%)					0.956
Yes	32 (23.4)	14 (13.1)	8 (15.4)	11 (20.4)	
No	105 (76.6)	93 (86.9)	44 (84.6)	43 (79.6)	
Short of breath, *n* (%)					0.165
Yes	18 (13.1)	3 (2.8)	3 (5.8)	1 (1.9)	
No	119 (86.9)	104 (97.2)	49 (94.2)	53 (98.1)	
Insomnia, *n* (%)					0.619
Yes	34 (24.8)	29 (27.1)	13 (25.0)	11 (20.4)	
No	103 (75.2)	78 (72.9)	39 (75.0)	43 (79.6)	
Abnormal sweating, *n* (%)					0.371
Yes	22 (16.1)	11 (10.3)	7 (13.5)	3 (5.56)	
No	115 (83.9)	96 (89.7)	45 (86.5)	51 (94.4)	
Fatigue, *n* (%)					0.341
Yes	30 (21.9)	24 (22.4)	10 (19.2)	8 (14.8)	
No	107 (78.1)	83 (77.6)	42 (80.8)	46 (85.2)	
Susceptible to colds (≥3times/year), *n* (%)					0.580
Yes	6 (4.4)	4 (3.7)	3 (5.8)	3 (5.6)	
No	131 (95.6)	103 (96.3)	49 (94.2)	51 (94.4)	

IQR, interquartile range.

a Mann–Whitney U test for continuous variables, the chi-square test for categorical variables; *p*-values for comparisons between training set and test set.

### 3.2 Acoustic feature selection

**Figure 3:**
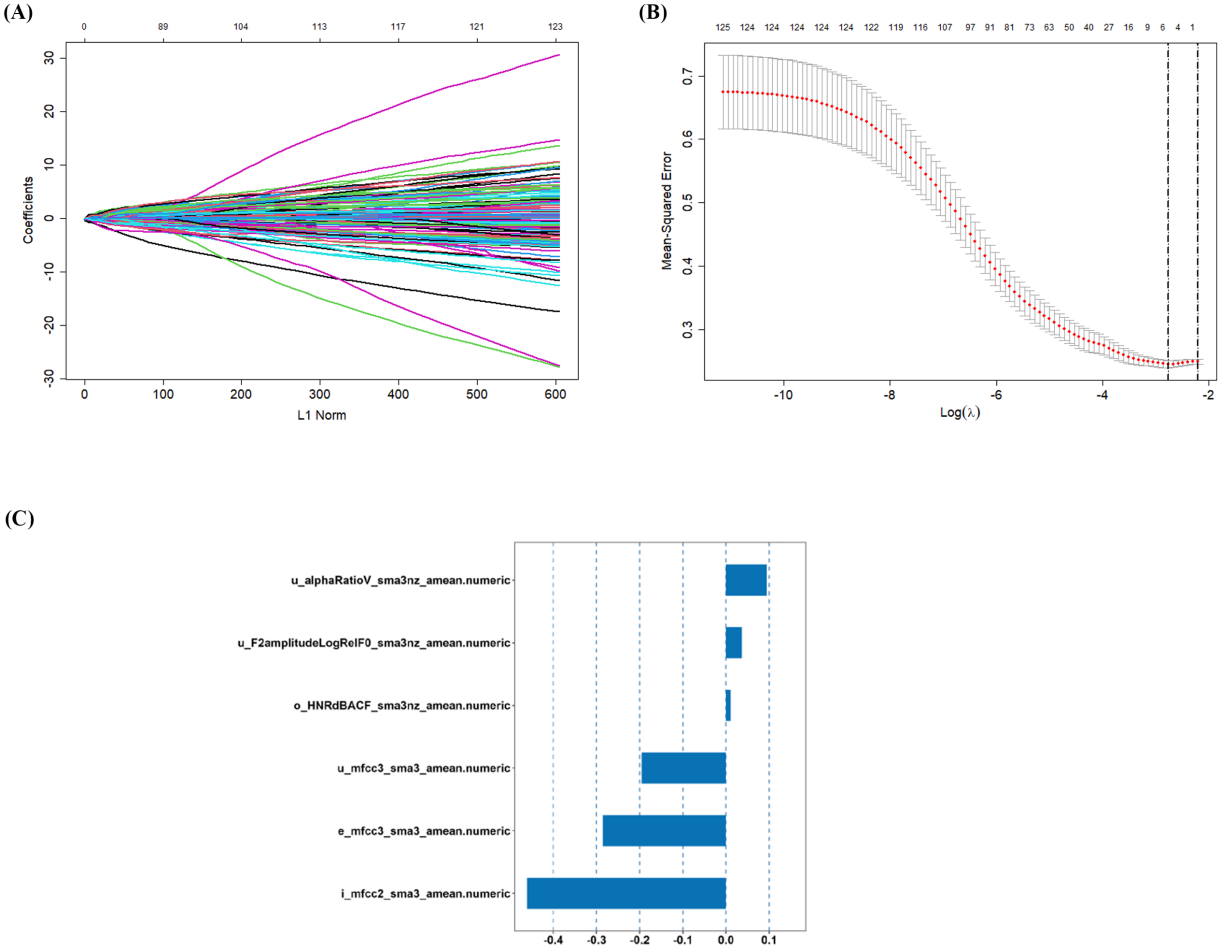
Screening of variables based on LASSO regression. **(A)** The variation characteristics of the coefficient of variables. Each curve in the figure represents the trajectory of the coefficient change for each independent variable. The *y*-axis represents the value of the coefficient, the lower horizontal axis represents the L1-norm value of the coefficient, and the upper horizontal axis represents the number of non-zero coefficients in the model at that time; **(B)** the selection process of the optimum value of the parameter *λ* in the LASSO regression model by 10-fold cross-validation method. In the LASSO model, the coefficient profiles of 125 acoustic features were drawn from the log (*λ*) sequence. Vertical dotted lines are drawn at the minimum mean square error (*λ* = 0.110) and the standard error of the minimum distance (*λ* = 0.063); **(C)** coefficients of six variables screened by LASSO regression. The text column on the left displayed the names of selected features. The bar chart on the right displayed the corresponding coefficient for each feature.

LASSO regression achieves automatic selection and comprehensive reduction of input features by incorporating an L1 regularization penalty term, which limits the magnitude of the regression coefficients (25). Thus, LASSO regression was used to screen acoustic features in the training set. In the iterative analysis, the 10-fold cross-validation method was applied, and a model with excellent performance and the minimum number of variables was obtained when *λ* = 0.063. Six optimal acoustic features, including e_mfcc3, i_mfcc2, o_HNR, u_alphaRatio, u_mfcc3, and u_F2 amplitude, were screened from 125 acoustic features, as shown in Figure 3.

### 3.3 Development of the prediction model based on acoustic–clinical features

**Figure 4:**
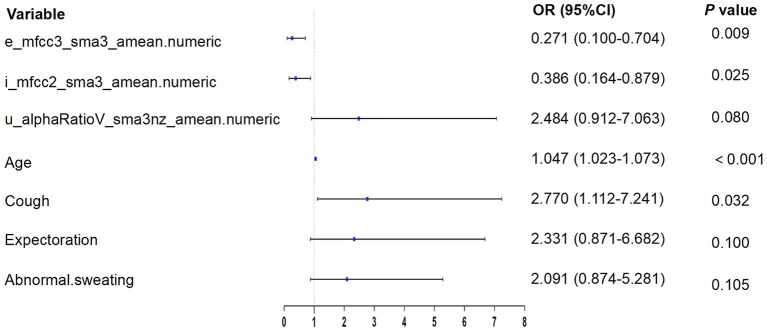
Forest plot for variable selection in multivariate logistic regression.

**Figure 5:**
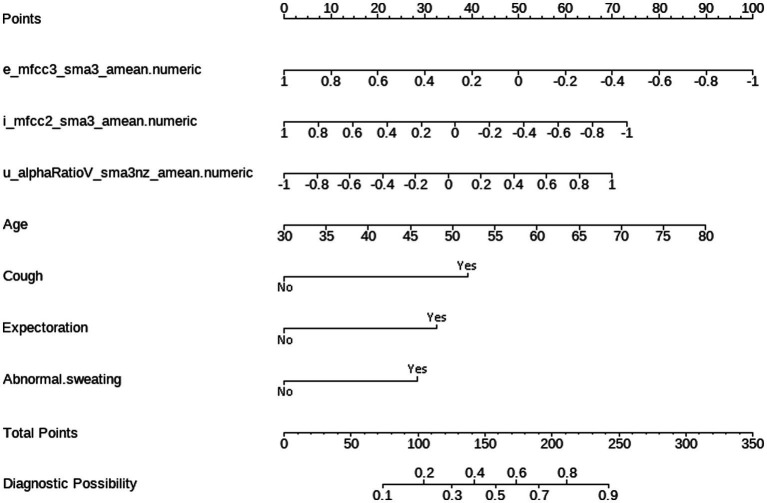
Nomogram for predicting lung cancer based on acoustic–clinical features. Incorporating acoustic features: e_mfcc3, i_mfcc2, u_alphaRatio, and clinical characteristics, including age, cough, expectoration, and abnormal sweating.

According to the results of multivariate logistic regression with backward elimination, the model containing e_mfcc3, i_mfcc2, u_alphaRatio, age, cough, expectoration, and abnormal sweating achieved minimal AIC value in the training cohort (Table 2; Figure 4). In addition, we assessed multicollinearity using variable inflation factors (VIF) and found that the VIF values for the selected variables were below 3. This model was presented as a nomogram for predicting lung cancer (Figure 5).

**Table 2:** Multivariate logistic regression analysis of acoustic–clinical features.

Variables	*β*-coefficient	SE	Adjusted OR (95% CI)	Z	*p*-value
(Intercept)	−2.349	0.711	0.095 (0.022–0.373)	−3.306	0.001
e_mfcc3_sma3_amean.numeric	−1.302	0.495	0.271 (0.100–0.704)	−2.629	0.009
i_mfcc2_sma3_amean.numeric	−0.951	0.426	0.386 (0.164–0.879)	−2.235	0.025
u_alphaRatioV_sma3nz_amean.numeric	0.910	0.520	2.484 (0.912–7.063)	1.750	0.080
Age	0.047	0.012	1.047 (1.023–1.073)	3.870	<0.001
Cough	1.019	0.474	2.770 (1.112–7.241)	2.149	0.032
Expectoration	0.846	0.514	2.331 (0.871–6.682)	1.647	0.100
Abnormal sweating	0.738	0.455	2.091 (0.874–5.281)	1.620	0.105

SE, standard error; OR, odds ratio; CI, confidence interval. The significant variables in the best-fit model were identified based on a multivariate logistic regression algorithm with a backward elimination method.

Interpretation method: Draw a vertical line for each variable of a subject, with the corresponding “points” representing the scores for that specific variable. The total score of the patient’s variables (total points) corresponds to the diagnostic possibility, which is the probability of lung cancer. For example, a 70-year-old subject with normalized voice features, specifically e_mfcc3, i_mfcc2, and u_alphaRatio values of −0.2, −0.1, and − 0.1 respectively, and who exhibits no symptoms of cough, phlegm, or abnormal sweating, would receive 70 points for age, 60 points for e_mfcc3, 40 points for i_mfcc2, 30 points for u_alphaRatio, and 0 points for the absence of cough, phlegm, and abnormal sweating symptoms. The total score would be 200 points, indicating a 75% predicted probability of lung cancer for this patient.

### 3.4 Evaluation and validation of the nomogram

**Figure 6:**
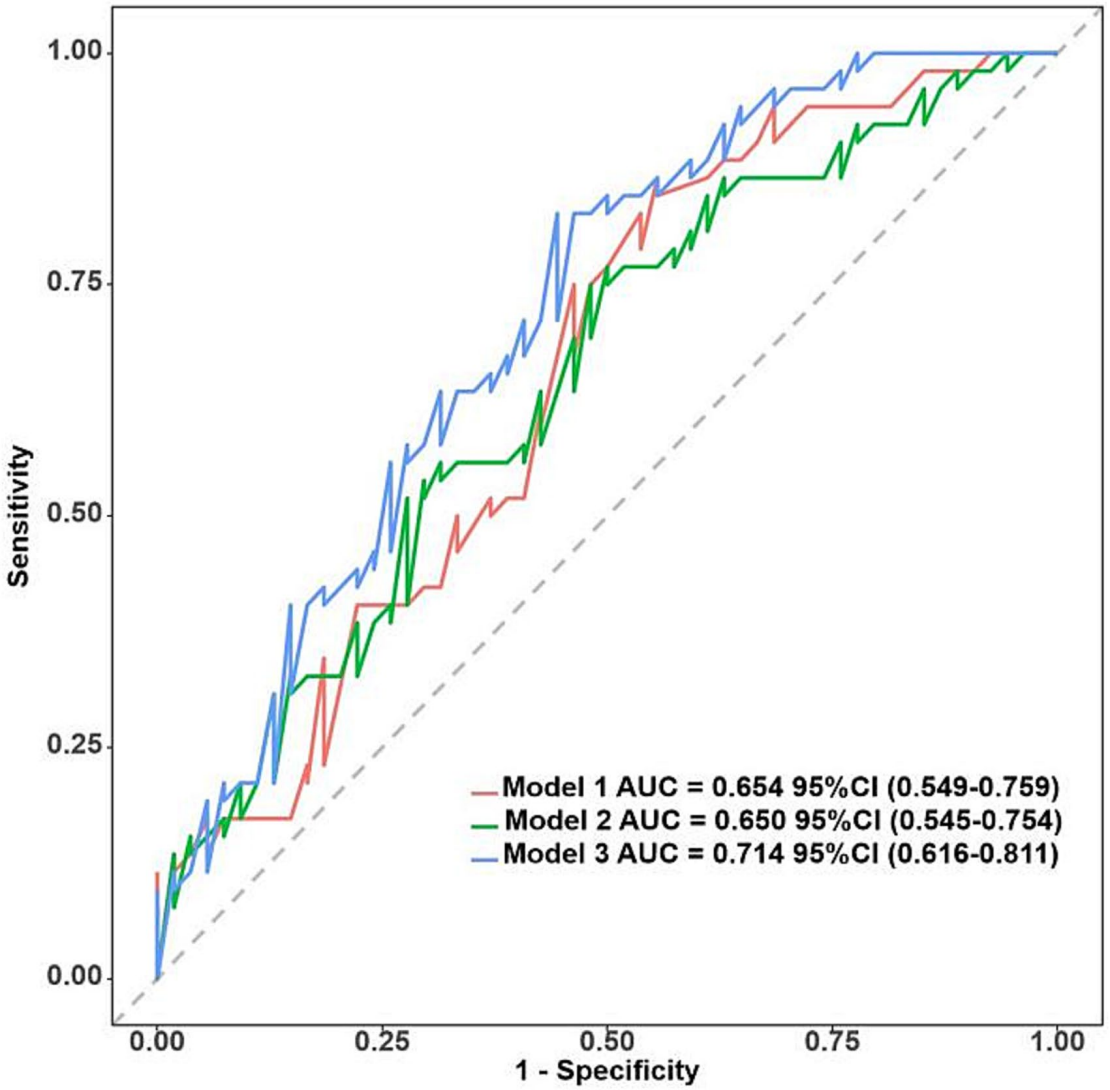
ROC curves for clinical characteristics (Model 1), acoustic features (Model 2), and acoustic–clinical model (Model 3) in the test set.

The acoustic–clinical nomogram model had a good discriminative ability with an AUC of 0.774 (95% CI: 0.716–0.832) in the training set. To validate the acoustic–clinical model, we conducted a comparative analysis of the ROC curves derived from multivariable regression models using the backward elimination method across three distinct feature sets: the first one was solely based on clinical characteristics (Model 1), the second one was solely based on acoustic features (Model 2), and the third one was a combined acoustic–clinical model (Model 3). As illustrated in Figure 6, in the test set, Model 3 exhibited a superior AUC of 0.714 (95% CI: 0.616–0.811) compared to Model 1 AUC (0.654, 95% CI: 0.549–0.759) and Model 2 (AUC: 0.650, 95% CI: 0.545–0.754). The performance evaluation of Model 3 in the test set revealed an overall accuracy of 0.642, a sensitivity of 0.673, and a specificity of 0.611.

**Figure 7:**
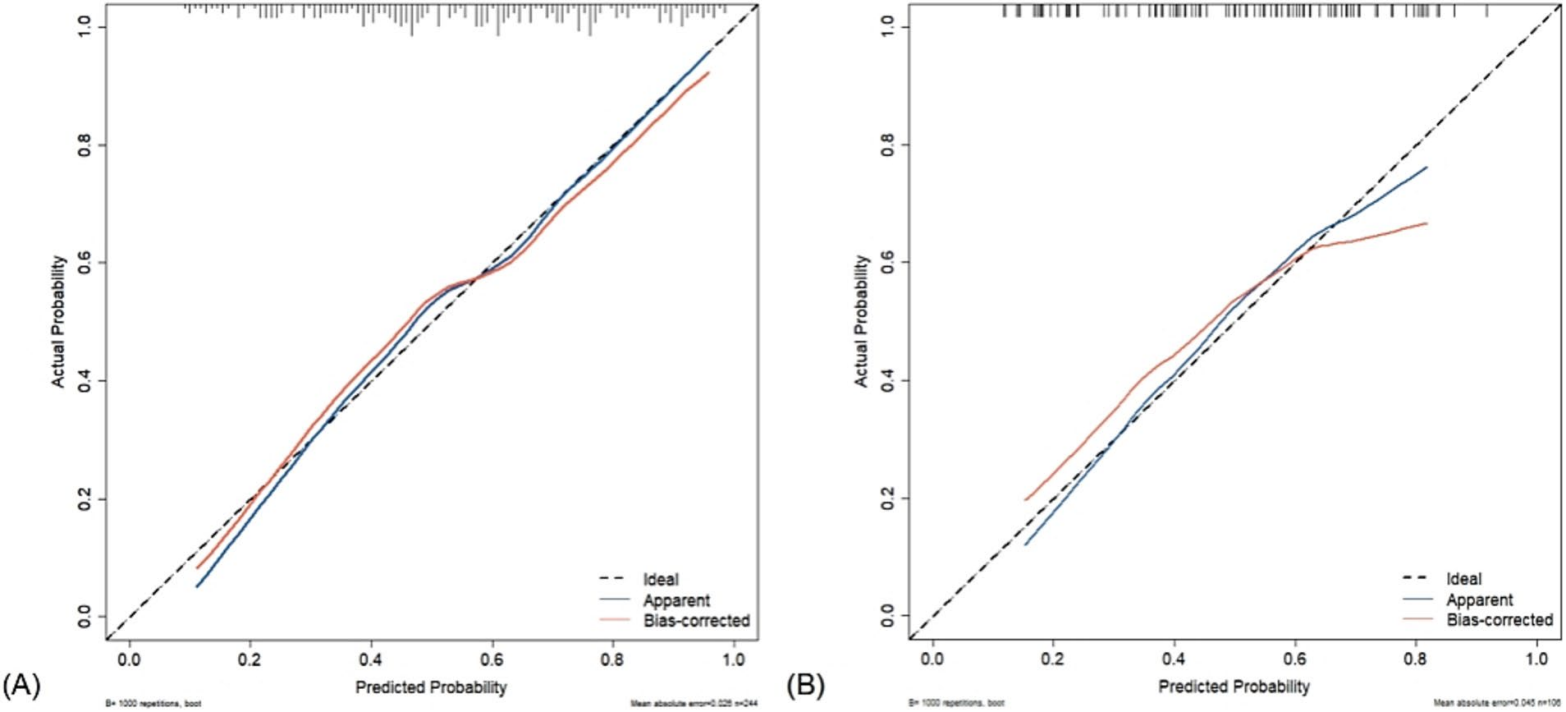
Calibration curve of acoustic–clinical nomogram. The dashed line represents the ideal model, where the predicted probability is the same as the actual probability. The blue line represents the actual performance of apparent accuracy, while the red line indicates the calibration curve of the corrected resampled estimation. **(A)** Training set and **(B)** test set.

The Hosmer–Lemeshow test revealed that the nomogram was well-fitting (training set: *χ*^2^ = 14.623, *p* = 0.067; test set: *χ*^2^ = 9.361, *p* = 0.313). The calibration curves obtained through the 1,000 bootstrap resamples method demonstrated good concordance with the ideal straight line, indicating the robust predictive performance of the nomogram model in relation to pathological outcomes (Figure 7).

**Figure 8:**
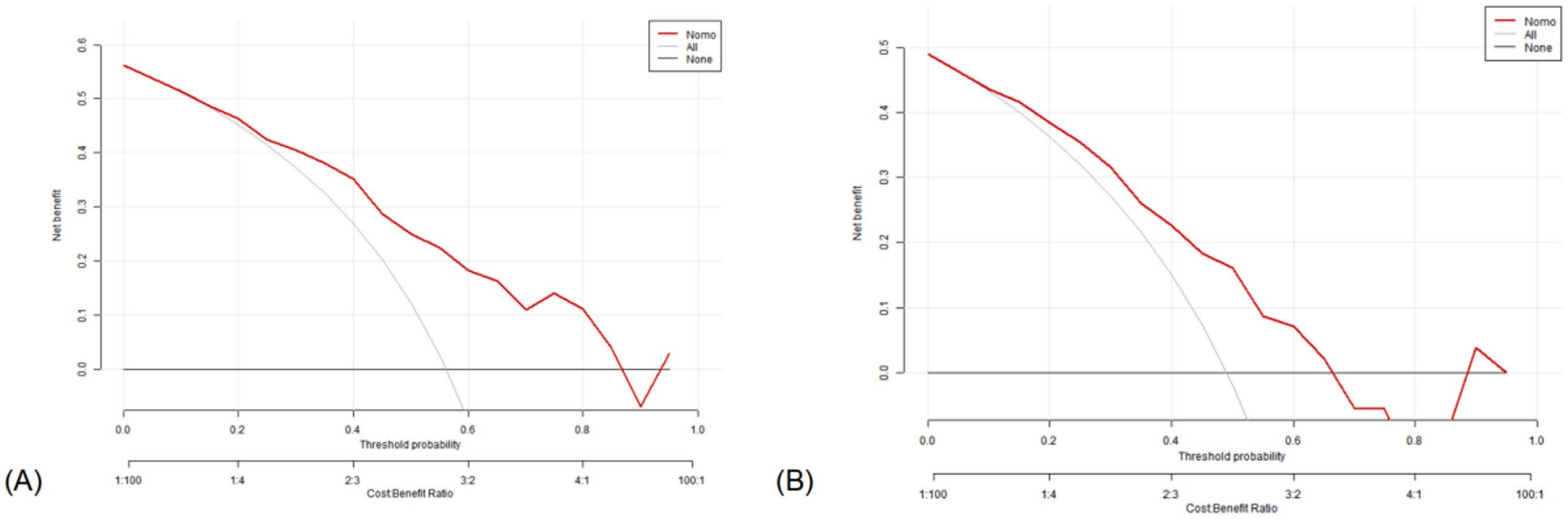
Decision curve analysis of the nomogram. X-axis line: threshold probability; Y-axis line: net benefit; gray line: hypothesis that all participants suffered from lung cancers; Black line: hypothesis that all participants did not apply the nomogram and the net benefit is zero; Red line: acoustic–clinical nomogram. **(A)** Training set and **(B)** test set.

In the training set, the DCA indicated that net benefits could be achieved with a threshold probability ranging from 15 to 87% (Figure 8A). Similarly, in the test set, the DCA demonstrated that net benefits were attainable within a threshold probability range of 10–65% (Figure 8B).

### 3.5 Comparison of predictive models using ML

**Figure 9:**
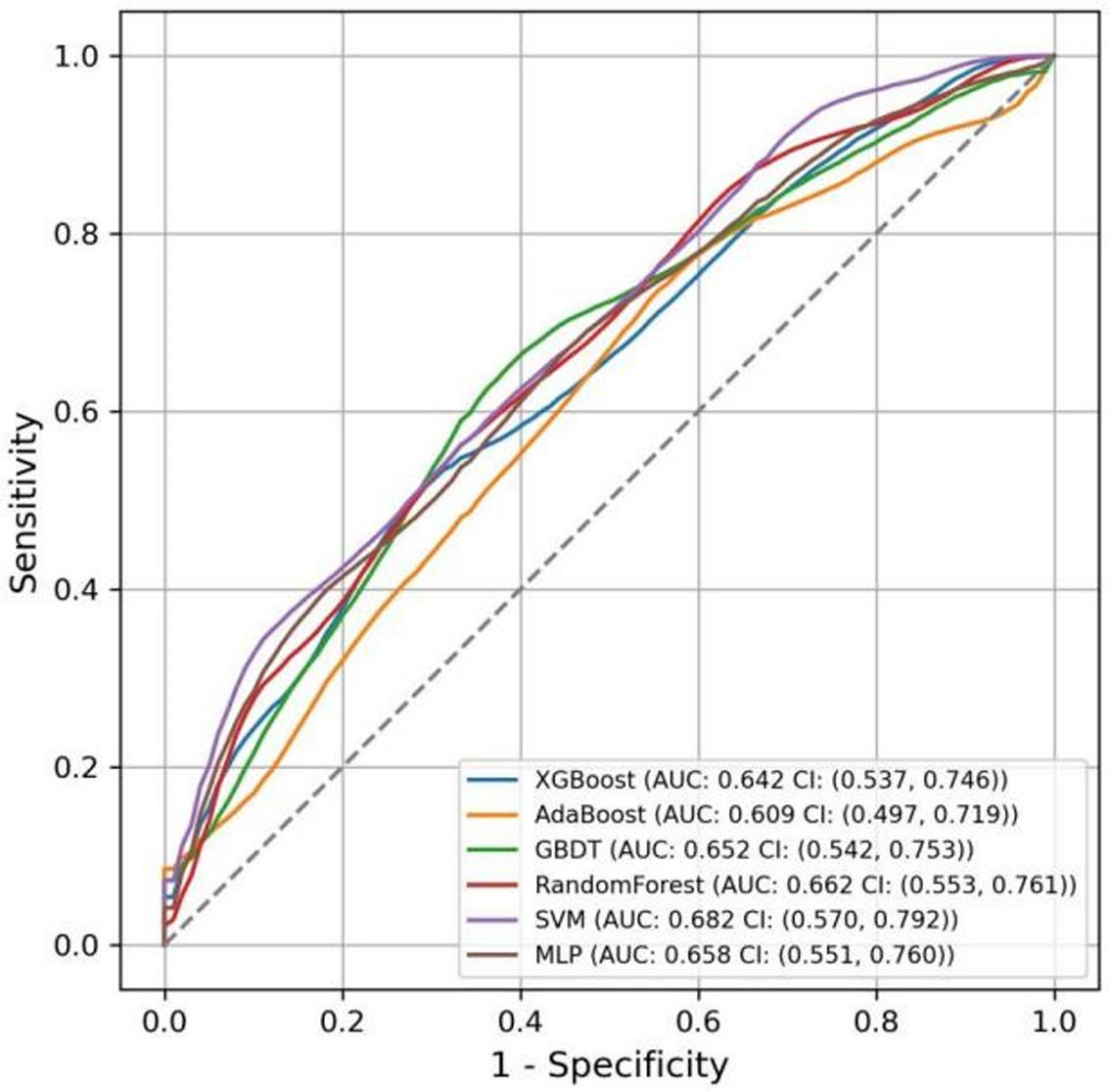
ROC curves for various ML models in the test set.

The AUC values for the models in the test set, obtained using various ML methods, are presented in Figure 9 as follows: XGBoost 0.642 (95% CI: 0.537–0.746), AdaBoost 0.609 (95% CI: 0.497–0.719), GBDT 0.652 (95% CI: 0.542–0.753), RF 0.662 (95% CI: 0.553–0.761), SVM 0.682 (95% CI: 0.570–0.792), and MLP 0.658 (95% CI: 0.551–0.760). Compared to six other ML models, the nomogram model exhibited the highest AUC.

The accuracy, sensitivity, specificity, and F1 score of various models were calculated, as reported in Table 3. The results indicated that the nomogram model excelled in accuracy and specificity, achieving 0.642 and 0.611, respectively, surpassing those of other models. AdaBoost and RF demonstrated the lowest accuracy at 0.581, and SVM showed the lowest specificity at 0.419. Regarding sensitivity, the SVM model exhibited superior performance, achieving a score of 0.774, while the nomogram model demonstrated the lowest sensitivity, scoring 0.673. For the F1 score, all models performed similarly, with the SVM model achieving the highest score of 0.663 and the AdaBoost model scoring the lowest at 0.623.

**Table 3:** Comparison of predictive performance among various ML models.

Model	AUC (95% CI)	Accuracy	Sensitivity	Specificity	F1 score
XGBoost	0.642 (0.537–0.746)	0.591	0.740	0.442	0.642
AdaBoost	0.609 (0.497–0.719)	0.581	0.679	0.477	0.623
GBDT	0.652 (0.542–0.753)	0.616	0.736	0.502	0.657
RF	0.662 (0.553–0.761)	0.581	0.699	0.461	0.625
SVM	0.682 (0.570–0.792)	0.601	0.774	0.419	0.663
MLP	0.658 (0.551–0.760)	0.630	0.696	0.559	0.652
Nomogram	0.714 (0.616–0.811)	0.642	0.673	0.611	0.648

AUC, area under the curve of ROC; XGBoost, extreme gradient boosting; AdaBoost, adaptive boosting; GBDT, gradient boosting decision tree; RF, random forest; SVM support vector machine; MLP, multilayer perceptron.

## 4 Discussion

Lung cancer is one of the most common malignant tumors (26). Due to individual differences among patients, lung cancer—which is prone to developing symptoms such as coughing, chest pain, and hemoptysis as the disease progresses (27)—is the most common neoplastic etiology underlying unilateral vocal fold paralysis (28). We developed a lung cancer voice database and developed and validated an accurate acoustic–clinical nomogram for predicting lung cancer. During the process of voice acquisition and speech recognition, it is susceptible to various factors (29, 30) such as device settings, the speaker’s accent, the distance of the mouth from the microphone, and background noise. To avoid the aforementioned interference, we implemented a standardized protocol for voice signal acquisition. Since vowels are produced without any physical obstruction in the vocal tract, allowing airflow from the lungs to pass through the glottis and strike the vocal chords, causing them to vibrate, we selected vowels as the pronunciation content and set fixed sampling rates, distances, and environmental scenarios.

The results of logistic regression analysis demonstrated that aging, cough, and acoustic features are independent risk factors for lung cancer. The acoustic–clinical nomogram model outperforms both the clinical characteristics model and the acoustic feature model in terms of discrimination performance. It also exhibited good calibration, indicating that the proposed nomogram may serve as an effective, non-invasive, and safe approach for lung cancer identification. During the diagnostic process for clinical lung cancer patients, heightened vigilance should be exercised for those who are elderly and exhibit symptoms such as cough, expectoration, abnormal sweating, and changes in voice quality, as these could indicate the presence of lung cancer.

MFCCs are coefficients formed through a linear transformation of logarithmic energy spectra based on the non-linear Mel scale of sound frequency, reflecting the auditory characteristics of the human ear, and are widely used in speech recognition (31). Alpha ratio refers to the ratio of the sum of energy between 50–1000 Hz and the sum of energy between 1–5 kHz, providing information on the relative intensity of low-frequency and high-frequency components in the voice signal. LASSO regression identified important features, specifically MFCC3, MFCC2, and alpha ratio, which were instrumental in the differential diagnosis of lung cancer. This finding suggested that patients with lung cancer may exhibit abnormalities in their voice timbre which these spectral features can capture. Modern medical researchers indicate that the main cause of voice alterations in lung cancer patients is the compression of the recurrent laryngeal nerve by advanced tumors (32). The main characteristics of this condition include changes in voice tone, hoarseness, or aphonia. Acoustically, when sound waves propagate through a medium and encounter obstacles while traveling, reflection can alter their energy, frequency, and wavelength. Consequently, when sound waves resonate in the lungs and encounter malignant tumors, differences in timbre may occur. Whether these alterations in timbre correlate with the size, number, and texture of the tumors remains to be further studied. The application of modern sensor technology for voice signal collection and conducting objective analysis may be more sensitive than the human ear at detecting voice changes in patients with lung cancer. Auscultation of voice using computer technology is not only cost-effective and easy to operate but also eliminates the need for other medical equipment that requires invasive procedures, thereby minimizing the risk of injury to patients.

This study used pathological findings as the gold standard and included individuals without pulmonary lesions to simulate real-world clinical scenarios. Our results suggested that the nomogram model demonstrated high predictive accuracy, calibration, and clinical applicability. Comparative analyses among various ML models revealed that the nomogram model surpassed others in AUC, accuracy, and specificity, thereby confirming its enhanced capability to identify individuals without lung cancer. While the SVM model achieved the highest sensitivity and F1 score of 0.774 and 0.663, respectively, indicating its proficiency in identifying lung cancer patients, its specificity was notably the lowest at 0.419. In contrast to complex ML models, the nomogram model exhibits simplicity, interpretability, and stability, which makes it more practically useful in clinical decision-making and a valuable screening tool for lung cancer.

Artificial intelligence technology has shown great potential and promising prospects, especially in the lung cancer screening, imaging examination, pathological testing, and biomarker detection (33). However, the potential of acoustic diagnosis for lung cancer remains largely unexplored. This study pioneered the innovative integration of voice acoustic features with clinical data, utilizing machine learning methods to construct model, laying the groundwork for an intelligent lung cancer diagnosis. Subsequently, we will explore the differences in acoustic features among lung cancer patients, individuals with benign lung nodules, and healthy controls and establish a multiclassification model to identify unique acoustic features that may differentiate these groups and potentially aid in early detection and diagnosis of lung cancer. In the future, applying acoustic diagnosis techniques will enhance lung cancer screening and diagnostic processes in regions where large-scale examinations are not feasible, offering high clinical application value and promising prospects for “internet plus healthcare”.

## 5 Limitations

Our study has some limitations. First, the voice is influenced by various factors such as physiological state, sex, age, mental status, and pathological conditions of speakers during sampling. Additionally, distinct speech representations exist among populations from different geographic regions, and this model is trained on Chinese language and may not be applicable to other languages. Secondly, the sample size of this study is relatively small, which may prevent the thorough exploration and evaluation of important subgroups, including sex, ethnicity, and others, and due to the absence of an external validation cohort in the study, the accuracy, generalizability, and transferability of the model need further verification. The sensitivity and specificity results of the proposed model are not yet satisfactory. In future research, we plan to increase the sample size and adopt resampling techniques to address the issue of data imbalance, enabling the model to better capture the characteristics of minority classes. In recent years, deep learning has demonstrated exceptional feature learning capabilities, high accuracy, and strong robustness in speech recognition. Given this, we will also attempt to apply deep learning algorithms, such as CNN, RNN, LSTM, and Transformer, to further optimize the model’s hyperparameters and enhance its generalization ability, aiming to improve its performance. Furthermore, current sample collection requires a quiet environment, yet noise is ubiquitous in daily life, which inevitably affects the conduct of large-scale sampling. As for remote healthcare, different recording devices introduce variations. Eliminating these discrepancies in natural settings poses a challenge for our research.

## 6 Conclusion

This study introduces a machine-learning approach for the disease prediction and screening of clinical lung cancer through voice signal feature analysis, which is non-contact, does not require specialist medical expertise or laboratory facilities, and can be deployed on inexpensive consumer hardware, such as a smartphone. Subsequent research studies are needed to collect voice information from various types and stages of lung cancer. By integrating acoustic diagnostic features with clinical information such as CT images, biochemical indicators, and lung function, deep learning algorithms will be applied to establish an intelligent diagnostic system and risk prediction model for lung cancer.

The nomogram model that we developed based on acoustic–clinical data shows good predictive performance—which is capable of predicting the clinical risks of lung cancer—and offers guidance for the screening of high-risk patients with lung cancer.

## Data Availability

The raw data supporting the conclusions of this article will be made available by the authors, without undue reservation.

## Glossary

LASSO: least absolute shrinkage and selection operator
ROC: receiver operating characteristic
AUC: area under the curve
DCA: decision curve analysis
CT: computed tomography
PNs: pulmonary nodules
MFCCs: Mel frequency cepstral coefficients
COVID-19: coronavirus disease 2019
LCa+: lung cancer group
LCa−: non-lung cancer group
PCM: pulse-code modulation
HNR: harmonics-to-noise ratio
IQR: interquartile range
SD: standard deviation
SE: standard error
OR: odds ratio
CI: confidence interval
ML: machine learning
XGBoost: extreme gradient boosting
AdaBoost: adaptive boosting
GBDT: gradient boosting decision tree
RF: random forest
SVM: support vector machine
MLP: multilayer perceptron
VIF: variable inflation factors
